# Whole-genome sequencing: identification of additional pathogenic variation across the genome

**DOI:** 10.1093/braincomms/fcab280

**Published:** 2021-11-19

**Authors:** James Dominic Mills, Sanjay M Sisodiya

**Affiliations:** 1 Department of Clinical and Experimental Epilepsy, UCL Queen Square Institute of Neurology, London, UK; 2 Chalfont Centre for Epilepsy, Buckinghamshire, UK; 3 Department of (Neuro)Pathology, Amsterdam UMC, University of Amsterdam, Amsterdam Neuroscience, Amsterdam, The Netherlands

## Abstract

This scientific commentary refers to ‘Genome sequencing identifies rare tandem repeat expansions and copy number variants in Lennox–Gastaut syndrome’, by Qaiser et al. (https://doi.org/10.1093/braincomms/fcab207).

**This scientific commentary refers to ‘Genome sequencing identifies rare tandem repeat expansions and copy number variants in Lennox–Gastaut syndrome’, by Qaiser et al. (https://doi.org/10.1093/braincomms/fcab207).**

Over the last decade, with improvements in DNA-sequencing technologies and advances in the handling and analysis of the resulting data, our understanding of the genetic architecture underlying developmental and epileptic encephalopathies has grown immensely.^1^ While current DNA-sequencing techniques allow for the entirety of the genome to be interrogated to the resolution of a single nucleotide, much of the progress has been driven through the use of targeted sequencing panels and whole-exome sequencing. Due to this, unsurprisingly, the focus has been predominately on causal single nucleotide variants that lay in the exons of protein-coding genes and have a direct impact on protein function. More recently, as DNA-sequencing has become more accessible, there has been a shift towards the utilization of whole-genome sequencing (WGS) techniques and the production of datasets further enriched in information. While this is a step in the right direction, many of the produced WGS datasets are not leveraged to their full potential that is, used to identify all potential pathogenic variation across the genome. Potentially pathogenic variation in non-coding regions, such as promoters or enhancers, or structural genomic variation including copy number variants or repeat expansions/tandem repeat (TR) expansions are often overlooked. Interestingly, despite being under-studied, there are well-known examples of variants in these elements being involved in human disease. These include some of the earliest discoveries of a human genetic disease such as the detection of a single nucleotide substitution in the promoter region of the haemoglobin subunit beta gene (encoding β-globin, a subunit of haemoglobin) that was found to reduce haemoglobin subunit beta gene expression, or the length of a TR expansion in the Huntingtin gene being negatively correlated with the age of disease onset.^2^^,^^3^

In the recent paper by Qaiser et al.^4^ in *Brain Communications*, blood-derived genomic DNA was extracted from a cohort of 30 adults with unexplained developmental and epileptic encephalopathies and subjected to WGS. The adults in this study had had prior genetic tests, including whole-exome sequencing, that were unremarkable or inconclusive. Qaiser et al.^4^ went beyond assessing the exons of protein-coding genes and analysed both the coding and non-coding genome for rare variants and alterations in the architecture of the genome using a variety of different tools and techniques. For single nucleotide variants and indels, the Broad Institutes’s Genome Analysis Toolkit Haplotype caller was used; copy number variants (CNVs) were investigated using the tool Estimation by Read Depth with Single-nucleotide variants and CNVnator; and TR using Expansionhunter Denovo, Tandem Repeat Finder and ExpansionHunter.^4^ Using this array of techniques, the group was able to provide a plausible genetic explanation for a further nine individuals in this cohort, including seven with potential pathogenic single nucleotide variants and two CNVs, and additionally, identified two TR expansions of unknown clinical significance. Thus demonstrating the usefulness of assessing the entirety of the genome of individuals with previously unresolved suspected genetic diseases.

Perhaps, most intriguing was the identification of TR expansions in the genes Disco Interacting Protein 2 Homolog B (*DIP2B*) and ATXN8 Opposite Strand LncRNA (*ATXN8OS*) in two patients with Lennox-Gastaut syndrome. Although these TR expansions could not be classified definitely as pathogenic, as there are no available guidelines to facilitate pathogenicity interpretation, these expansions were in the size range of disease-causing expansions seen in other conditions. Furthermore, to the best of the authors’ knowledge, this is the first time TR expansion has been reported in Lennox-Gastaut syndrome. This finding raises the question: is this due to TR expansions being rare in Lennox-Gastaut syndrome or simply because TR expansions are rarely looked for in Lennox-Gastaut syndrome patients? As only six patients with Lennox-Gastaut syndrome were included in this study, of which two had TR expansions, it is possible that the latter is true, but we need to acknowledge that the sample size overall is small. Nevertheless, this intriguing finding raises the question of how important TR might be for other developmental and epileptic encephalopathies or other forms of human genetic disease? How much of our understanding of the pathogenicity and strategies for potential treatments are being missed by the focus on protein-coding genes in general? This possibility is further underlined by well-known examples of causal TR expansions in other human diseases such as Huntington’s disease and amyotrophic lateral sclerosis^3^^,^^5^ as well as some other forms of epilepsy, including such as some progressive myoclonic epilepsies^6^ and familial adult myoclonic epilepsy.^7^ Despite this, analyses for TR expansions are not routine in the clinical assessment of potential genetic epilepsies. This concept can be further extended to CNVs, identified in two of the individuals, including in one of the six people with Lennox-Gastaut syndrome in the paper by Qaiser et al.^4^ Recently, the Lal laboratory identified specific copy number burdens for epilepsy subtypes, showing that 1.5–3% of people with common epilepsy types carry epilepsy-associated CNVs,^8^ suggesting that CNVs are not a rare occurrence in genetic epilepsies. However, it must be noted, that while pathogenic structural variations in the genome may be relatively common, this field of study is still comparatively young and is hampered by the lack of experimental validation of potentially pathogenic structural variants and the lack of standardized calling and interpretation guidelines for declaring variants pathogenic. The same shortcomings can also be seen in the study generally of non-coding variation, where our understanding of genetic variation influencing gene expression or regulation lags behind our understanding of the impact of variants on the protein-coding genes.

The research performed by Qaiser et al.^4^ demonstrates a contemporary framework for performing genetic studies using WGS data: utilizing a variety of tools, assessing the whole genome, and not stopping the analysis on finding the first putatively culpable variant. Furthermore, some variants were experimentally validated using the appropriate wet-laboratory techniques. But perhaps most interestingly, while a variety of tools were used to assess the whole genomes presented, there remain other tools and additional analyses that could be applied, for example, searching for mosaic variants.^9^ And this perhaps highlights some of the most exciting aspects of producing WGS data: the whole genome is well covered, so that advantages of next-generation sequencing data in general can be amplified: data can be stored for the long term and re-analysed as new tools are created, and as genome annotations are improved; or shared with other researchers who have different research questions, or combined with other datasets to produce larger, more powerful datasets. Indeed, these ideas are fundamental and central to the European Commission-funded research project ‘Solve-RD-solving the unsolved rare disease’ (solve-rd.eu) which focuses on collecting unsolved cases with available whole-exome sequencing and WGS data from several genetic laboratories and assessing these data using the most up-to-date tools and genome annotations. We currently find ourselves in the midst of the information age, and thus far technological advances have dramatically improved our understanding of the link between genome and phenotype. As we continue to progress, we will continue to come up with more and more genetic explanations for, or contributions to, human disease, which will no doubt improve our ability to manage and treat people with epilepsy.
